# The usefulness of optics-based courses for optometry and vision science alumni: a cross-sectional online survey

**DOI:** 10.1080/07853890.2022.2076899

**Published:** 2022-05-18

**Authors:** Mohammad A. Alebrahim, May M. Bakkar

**Affiliations:** Faculty of Applied Medical Sciences, Department of Allied Medical Sciences, Jordan University of Science and Technology, Irbid, Jordan

**Keywords:** Optometry alumni, optometry curricula, optometrists' perceptions, geometrical and physical optics, visual optics

## Abstract

**Objectives:**

A well-balanced curriculum is critical for ensuring that students graduate with the necessary skills. There is growing interest in evaluating the functional value of non-clinical courses compared to clinical courses. The purpose of this study was to evaluate the views of optometry and vision science professionals on the utility and relevance of optics-based courses.

**Methods:**

A web-based survey was designed to assess the perceived significance of the optics-based courses. All respondents were alumni of two universities in Jordan that offer an undergraduate degree in optometry. The survey included questions about the professional relevance of optics courses. Respondents were asked to rate several statements related to the relevance and importance of optics courses in current optometry practice on a 5-point Likert scale.

**Results:**

In total, 205 respondents completed the online survey. There were 161 (78.5%) women and 44 (21.5%) men, with an average age of 28.76 (± 4.86) years. Overall, respondents rated the perceived usefulness of optics courses for their careers with a mean collective value of 20.78 out of a possible 30 points. Female alumni perceived the courses as significantly more useful in their workplace than male alumni did (*p* = .020). Optometrists in different age groups perceived usefulness differently (*p* = .001). Alumni who preferred to learn these courses on-site and by hybrid methods perceived optics courses as significantly more useful than those who preferred to learn online (*p* = .006 and *p* < .001, respectively).

**Conclusions:**

The perceived importance of optics-based courses varied according to several factors. However, in general, these courses were deemed helpful in terms of curricular content and practical relevance to practice, regardless of specialisation or the workplace.

**Key messages**
1. In terms of the value offered to the professional competencies that an optometrist needs, the practical utility of optics courses in optometry curriculums is contested.
2. Optometrists' perceptions of optics courses were examined using a cross-sectional survey.
3. Respondents rated the perceived usefulness of optics courses for their career, with a mean collective value of 20.78 out 30 points.
4. The highest percentage of respondents in this study expressed their preference for the face-to-face learning method rather than the hybrid method.
5. Male optometrists perceived learning optics courses as significantly less helpful than female optometrists.

## Introduction

Optometry is a healthcare profession that focuses on the evaluation of vision and the visual system to detect functional or anatomical abnormalities [1]. To become optometrists, students must be qualified to perform such evaluations and participate in various learning activities related to optics, the human body, health, and eyes to become optometrists [2].

In healthcare education, learning goals focus on mastering competencies in patient-centered care, working in interdisciplinary teams, evidence-based practice, regular quality improvement, and the use of information technology to support decision-making. As optometrists are classified as primary healthcare professionals, competencies in the field of optometry include general competencies expected from healthcare professionals, as well as “ability to do” competencies in specific areas related to optometry such as visual function and ametropia, binocular vision, ocular examination methods and prescription of optical aids, contact lenses, familiarity with ocular pathologies, and ability to take appropriate actions for referral and follow-up of visual function evaluation [3–6].

Optometry curricula worldwide include medical sciences courses (e.g. pathology, neuroscience, and microbiology) and courses related to optics [7–10]. Optics is a branch of physics that deals with studying the properties of light and its behaviour, such as interaction with matter, reflection, refraction, interference, diffraction, and polarisation [11,12]. Optics courses in the optometry curriculum aim to provide students with primary knowledge of two main fields, geometrical optics and visual optics, which form the basis for ocular physiology, contact lenses, low vision, and ophthalmic instrumentation [13–19].

Recently, there has been a significant interest in examining the impact of different components of the optometry curriculum on actual practice. Efforts are also being made to modernise optometry education to be more patient-centric and yield the highest value in terms of professional development [20,21]. In this context of multiple alternatives, it is necessary to carefully examine the functional utility of each course offered in the optometry curriculum [22].

Carneiro *et al.* conducted a nationwide questionnaire survey in Portugal to examine the general competencies and training needs of optometrists [5]. Their survey revealed that courses such as applied ocular pharmacology, applied ocular pharmacology, and prismatic prescription are among the areas of training need. These authors also highlighted that the scope of practice of optometrists in Portugal is considerably different from that in countries such as the United Kingdom, Australia, and the United States [23]. Diversity in optometric education has been reported in China [14] and in several countries, and a gap has been identified between the content of the optometry curriculum and the functional skills required in optometry practice [24,25]. These discrepancies make it necessary to re-examine the relevance of components of the optometry curriculum to the current professional needs of optometry students. Furthermore, as optometry education has shifted from the domain of physical science to medical science, there is an ongoing debate about reducing the components of optics and increasing the number of courses related to medicine [26]. However, as Atchison argues, complete knowledge of optics is necessary to prescribe suitable ocular devices to patients [27]. To gauge the perceived value of optics, a survey was conducted among 37 optometrist alumni of the University of Manchester [17]. The survey revealed that most respondents acknowledged the importance of optics as a subject, although there is a need to make optics more relevant to the current needs. However, no other study has evaluated the relevance of optics-based courses in optometry practice.

The objective of the current study was to evaluate the views of optometry and vision science graduates in Jordan, who have experienced the utility and relevance of optics-based courses in the optometry and vision science profession. To this end, an online survey was conducted with Jordanian optometry alumni to assess the perceived usefulness of optics courses and their relevance to optometry practice.

## Methods

### Study design

#### Type of research

A cross-sectional online survey was conducted between November 1, 2020 and December 31, 2020.

#### Study setting

Jordan is a middle-income country in the Middle Eastern country. Optometry education in Jordan is based primarily on the Global Competency-Based Model of Scope of Practice in Optometry [3]. Jordan's education programs meet level 3 World Council of Optometry (WCO) competencies, and every year in Jordan, approximately 35 students graduate in optometry.

#### Sampling/respondents

The target population included Jordanian optometry alumni working in different fields of optometry.

#### Ethical consideration

This study was approved by the Institutional Review Board (IRB) of the Jordan University of Science and Technology (Irbid, Jordan). Ethical reference number: 129/136/2020. The purpose and importance of the study were explained to the respondents. Informed consent was obtained electronically from all the respondents before proceeding with the survey questions. Participation in this study was voluntary. To ensure privacy and confidentiality, the anonymity of the personal information of the respondents was preserved. The study protocol complied with the provisions of the Declaration of Helsinki regarding research involving human participants.

### Course information

Two universities in Jordan offer an undergraduate degree in optometry: Jordan University of Science and Technology (JUST)-Irbid and Al-Ahliyya Amman University (AAU), Amman. Optics courses are included as core elements of optometry curricula at both universities. The study participants studied two optics courses during the first and second year of their university education: i) geometrical and physical optics, and ii) visual optics.

The geometrical and physical optics course involves three credit hours covered in the second semester of the first academic year; the semester contains 14 weeks with 42 lectures. Learning objectives (weightage%) are the nature of light, rays, waves, and particles, the dual nature of light (5%), specular and diffuse reflection (5%), Huygens' principle (5%), laws of reflection and refraction (10%), application of the law of reflection(10%), Snell's law and its application (10%), thin and thick lenses (10%), common vision defects (10%) composition and function of the microscope (15%) interference and diffraction of light (10%) and polarisation of light (10%). This assessment involves three exams. The first is conducted in the fifth week, the second is conducted in the ninth week, and the last is conducted at the end of the semester.

The visual optics course involves two credit hours covered in the first semester of the second academic year, with two hours per week and 14 weeks with 28 lectures. The learning objectives (%weightage) are optics of the human eye (30%), calculation of the imaging properties of eyes corrected by spectacles or contact lenses (30%), properties of lenses (20%), and properties of prisms (20%). The learning outcome is to "outline the optical characteristics of the eye in relation to the visual performance and the optics of corrected lenses." This assessment involves three exams. The first is conducted in the fifth week, the second in the tenth week, and the last is conducted at the end of the semester.

### Conceptual framework

The effectiveness of the academic program was gauged based on the Kirkpatrick framework [27,28]. This framework is widely used to examine the effectiveness of educational programs, mainly because of its ease of use in establishing a method to assess training results and simplifying a complicated evaluation procedure [27,29]. This framework comprises four levels ranging from the relevance of the program to the needs of the respondents (Level 1) to the real difference that the program has made in the health care outcome (Level 4). Level 2 is related to the knowledge that the practitioner gained from the program, and Level 3 indicates the application of the knowledge gained during the program to practice.

### Measurement and data collection

A web-based survey was designed in accordance with a previous study [17], expanding on selected elements considered to play a significant role in assessing the usefulness and relevance of optical-based courses for optometry alumnus practice.

The survey was written in English because it is a medium of instruction at Jordanian universities. The questions included in the survey were suggested and reviewed by the research team and then reviewed by a focus group consisting of three optometrists and one academic to ensure ease of understanding and appropriateness. The final version of the survey was administered using Google Forms (Google Inc., CA, USA).

The final version of the web survey was shared online *via* Social, WhatsApp, and LinkedIn platforms for six weeks between November 1, 2020 and December 31, 2020. The average time required to complete the survey was five minutes. The initial part of the survey sought sociodemographic information, such as age, sex, year of graduation, primary specialty, and workplace. The survey also consisted of questions on ophthalmic procedures that the respondents provide routinely in their practice. Subsequently, respondents were asked to express their agreement or otherwise on several statements related to the relevance and importance of optics courses to current optometry practice on a 5-point Likert scale (1 = strongly disagree, 2: disagree, 3: neutral, 4: agree, 5: strongly agree). The survey also included questions related to the demographics of the respondents, the ophthalmic procedures they routinely performed, and their views on the relevance of the optics courses they had been taught during the undergraduate study to their current career in optometry.

### Data analysis

Data were analysed using the Statistical Package for Social Sciences software version 21 (SPSS, International Business Machine Corp. IBM, Chicago, IL, USA). FACTOR statistical analysis software was used for quantitative data analysis [30]. The alpha significance level was set at *p* < .05. Means and standard deviations were calculated to describe the continuously measured variables. Categorical variables were described as frequencies and percentages. Data were tested for normality using a histogram eyeball test and Kolmogorov-Smirnov statistical test. Cronbach's alpha test was used to assess the reliability of the optometrists' six perceived indicators of usefulness and satisfaction with optics learning materials. Principal component analysis with factor analysis was considered a dimension-reduction technique to reduce the measured indicators of the usefulness of optometrists' satisfaction with optometry optics courses into a more straightforward and meaningful construct. Multivariate linear regression analysis was used to determine whether a specific factor, such as age, sex, or specialty, affected the perceived usefulness of optics courses. Furthermore, using parallel analysis, factor analysis was applied to test whether the six satisfaction indicators with the optics courses comprised unidimensional scores. Additionally, for unidimensionality, Unico (Unidimensional Congruence), mean IREAL (Mean of Item Residual Absolute Loadings), and ECV (Explained Common Variance) tests were applied.

The relative importance index (RII) was also calculated to reveal the relevance of these courses to subsequent careers of optometrists. The formula used to calculate the RII was provided by Holt, 2015 [31], and an RII, a 50% item, was considered substantive. The bivariate independent sample t-test and the one-way ANOVA test were used to assess the statistical significance of the mean difference in the perceived relevance of optics courses across the levels of the categorical characteristics of the respondent optometrists when an unequal variance was met, an adjusted statistical test statistic and associated (*P*-values) for both the unpaired *t*-test and the one-way ANOVA tests. Multivariate linear regression analysis was used to assess the combined and individual associations of the sociodemographic and professional characteristics of optometrists.

## Results

### Sociodemographic characteristics

We received 205 valid responses. There were 161 (78.5%) women and 44 (21.5%) men, with an average age of 28.76 (± 4.86) years. Of these, 39% were between the ages of 26 and 30, and 8.3% were under the age of 36. Approximately half of the respondents (51.1%) reported that they worked in optical shops, with refraction being the most common specialty (Table 1).

**Table 1.** Sociodemographic and professional characteristics of the study population (*N* = 205).

	Frequency	Percentage
Gender		
Female	161	78.5
Male	44	21.5
Age (years), mean (SD)		28.76 (4.86)
Age group		
21–25 years	57	27.8
26–30 years	81	39.5
31–35 years	50	24.4
≥36 years	17	8.3
Graduation year		
Before 2005	14	6.8
2006–2010	47	22.9
2011–2015	58	28.3
After 2015	86	42
Current specialty		
Academia	20	9.8
Contact lenses	44	21.5
Low vision	58	28.3
Orthoptics	12	5.9
Pediatrics	26	12.7
Refraction	45	22
Current working place		
Other / Unemployed	5	2.4
Hospital	26	12.7
Optics shop	105	51.2
Academia	13	6.3
Eye centre	56	27.3

### Perceived usefulness of optics courses

#### Principal component analysis

Factor analysis with principal component analysis and parallel analysis considering six indicators was applied to reveal the usefulness of optics courses, as shown in Table 2. All six optics course satisfaction indicators were significantly loaded (well above 0.60) into the single latent perceived usefulness of the optics course factor. The sample adequacy K-M-O (Kaplan-Meyer-Olkin) showed that the analysed sample of optometrists was sufficient, KM-*O* = 0.82, and the determinant index was equal to 0.068. Bartlett's test of sphericity, *χ*^2^(15) = 539.4, *p < .001*, showed that the correlation matrix of these six indicators was analysable with factor analysis and did not show unwanted collinearity. However, parallel analysis and unidimensionality tests (Unico = 0.97, ECV = 0.851, and M-IREAL = 0.253), along with scree plot analysis, all reflected the agreement that these six indicators comprised a single latent factor. The single latent factor solution explained 58.1% of the shared correlations (covariances) between the indicators of perceived satisfaction of optometrists with aspects of optics, courses, and their relevance to the optometrists' profession. Cronbach's alpha test showed that the six satisfaction indicators of the optics course were measured reliably, with Cronbach's alpha of 0.85. Six indicators of satisfaction of optometrists with optics courses were added, comprising a total score (6–30 points) denoting the perceived usefulness of optics courses, with a higher score indicating the greater perceived usefulness of the college courses for the optometry profession, which was analysed as a dependent outcome variable in bivariate and multivariate analyzes. The total score was based on the factor analysis of the six indicators, which were measured using a Likert-like scale (1 = strongly disagree to 5= strongly agree).

**Table 2.** Principal components analysis matrix regarding the perception of optometrists about the usefulness of optics courses.

	Perceived usefulness of optics courses
Q.1 What I learned during my studies in the optics courses has been useful in my practice.	0.853
Q.2 The educational material in these courses I received was relevant to my career.	0.848
Q.3 I enjoyed the topics I received in these courses	0.771
Q.4 I believe that optics courses should be taught in the optometry curriculum	0.767
Q.5 The practitioner licence exam included questions from many areas of these optics courses	0.667
Q.6 teaching staff (doctors/TAs) were capable of teaching the optics courses	0.643

Extraction Method: Principal Component Analysis. No rotation was Needed for the single latent factor.

#### Optometrists' perceptions regarding optics courses

The optometrists' overall mean perception of the usefulness of the optics courses for their careers was rated with a mean collective rating of 20.75 out of a maximum of 30 points. This is equivalent to 69.2% ((20.78/30) × 100), indicating the substantive perceived usefulness of the optics courses, as summarised in Table 3.

**Table 3.** Optometrists' perceptions regarding optics courses. *N* = 205.

	Mean (SD)	Disagree *n* (%)	Neutral *n* (%)	Agree *n* (%)	RII	*Rank*
Overall (total) perceived usefulness of the Optics courses (5–30 maximum points)	20.75 (4.78)	_	_	_	_	*_*
What I learned during my studies in the optics courses has been useful in my practice.	3.50 (0.99)	36 (17.6)	29 (14.1)	140 (68.3)	70.0	*4*
The educational material in these courses I received was relevant to my career.	3.52 (1.10)	39 (19)	36 (17.6)	130 (63.4)	70.4	*3*
I enjoyed the topics I received in these courses.	3.35 (1.22)	52 (25.4)	36 (17.6)	117 (57.1)	67.0	*5*
I believe that optics physics courses should be taught in the optometry curriculum	3.77 (0.94)	25 (12.2)	35 (17.1)	145 (70.7)	75.3	*1*
The teaching staff (doctors/TAs) were capable of teaching the optics courses	3.10 (1.20)	69 (33.7)	46 (22.4)	90 (43.6)	61.4	*6*
The practitioner licence exam included questions from many areas of these optics courses.	3.54 (0.91)	24 (11.7)	57 (27.8)	124 (60.5)	70.8	*2*

#### Bivariate and multivariate analysis

Table 4 presents the bivariate analysis of the means of the optometrists' perceptions of the usefulness of the optics courses. According to an unpaired sample *t*-test, the results showed that female optometrists perceived optic courses as significantly more useful (mean = 21.16) than male optometrists (mean = 4.59), *p* = .020. Furthermore, Welch's adjusted one-way ANOVA test showed that optometrists in different age groups perceived the usefulness of optic courses differently (*f* (3,59.30) = 6.45, *p* = .001). In contrast, a post hoc Games-Howell pairwise comparison test reported that optometrists aged 26 to 30 years perceived optics courses as significantly more useful (mean = 22.16) than those aged 31 to 35 years (mean = 19.22, *p* = .002). Optometrists between 26 and 30 years perceived optic courses as significantly more useful than those aged < 36 years (*p* = .039). However, the mean perception of the usefulness of optics courses by other optometrists in different age groups did not necessarily differ significantly when pairwise comparisons were made.

**Table 4.** Bivariate analysis of the optometrists' mean perceived usefulness of optics courses. *N* = 205.

	Mean (SD) perceived usefulness of optics courses	test statistic	*p*-value
Sex			
Female	21.16 (4.59)	*t* (203) = 2.34	.020
Male	19.27 (5.23)		
Age group			
21–25 years	21.14 (4.61)	*f* (3,59.30) = 6.45	.001
26–30 years	22.16 (3.77)		
31–35 years	19.22 (4.80)		
≥36 years	17.24 (6.68)		
Graduation year			
Before 2005	18.57 (6.91)	*f* (3,50.74) = 9.4	<.001
2006–2010	18.19 (4.92)		
2011–2015	22.47 (3.16)		
After 2015	21.35 (4.58)		
Current specialty			
Academia	21.75 (4.34)	*f* (5,199) = 1.60	.160
Contact lenses	20.39 (4.90)		
Low vision	21.31 (4.74)		
Orthoptics	18.17 (5.62)		
Pediatrics	21.85 (3.48)		
Refraction	20.00 (5.14)		
Current working place			
Other/unemployed	20.20 (6.22)	*f* (4,200) = 0.62	.650
Hospital	21.10 (4.54)		
Optics shop	20.35 (5.10)		
Academia	22.31 (4.87)		
Eye centre	21.04 (4.21)		
Which do you think is the best method for learning optics courses?			
Online	16.79 (5.36)	*f* (2,202) = 10.30	<.001
Hybrid course	21.00 (4.23)		
Classroom	21.37 (4.54)		

Furthermore, Welch's adjusted one-way ANOVA test showed that the optometrist graduation period converged significantly with their mean perception of the usefulness of optics courses, *f*(3,50.74)=9.4, *p < .001*. The Games-Howell post hoc pairwise comparison test revealed that optometrists who graduated between 2006 and 2010 perceived optics courses as significantly less useful (mean = 18.19) than those who graduated between 2011 and 2015 (Mean = 22.47), *p < .001*, and those who graduated after 2015 (mean = 21.35), *p* = .003. According to the one-way ANOVA test, optometrists' preference for learning methods converged significantly with the mean of their perception of the usefulness of optics courses' usefulness, *p < .001*. The highest percentage of respondents in this study (66.8%) expressed their preference for the face-to-face learning method rather than hybrid (a mixture of face-to-face and e-learning, 21.5%) or distance e-learning methods (11.7%). However, Bonferroni's analysis by adjusting the post-hoc pairwise comparison test showed that optometrists who preferred the online method perceived optics courses as significantly less useful to their work than optometrists who preferred hybrid and hybrid on-site methods, *P ≤ .001*. However, the other pairwise comparisons between optometrists with different graduation periods were not statistically significant. The specialty and work environment of the study population, according to this investigation, did not converge significantly with the respective means of their perceptions of the usefulness of optics courses.

Multivariate linear regression analysis was then used to determine the findings of the bivariate analysis, as shown in Table 5. The results showed that at least one of the characteristics tested had a statistically significant multivariate association with their perception of the usefulness of learning optics courses, *f*(7,197) = 7.20, *p < .001*. In detail, the resulting findings of the multivariate model revealed that the age, specialty, and work sector of optometrists did not statistically significantly converge with the mean of their perception of the usefulness of the optics courses. However, the analysis model indicated that male optometrists perceived learning optics courses as significantly less useful than did female optometrists (beta = −1.72, *p* = .023). The analysis model also showed that optometrists who graduated after 2011 perceived learning optics courses as significantly more useful on average than those who graduated before 2011 (beta = 3.482, *p = .023*). However, optometrists who preferred to learn these courses both on-site and through hybrid methods perceived learning optics courses as significantly more useful than those who preferred to learn through online methods, *p = .006* and *p < .001*, respectively.

**Table 5.** Multivariate Linear Regression analysis explaining the optometrists' perceived usefulness of optics courses. *N* = 205.

		95% C. I for beta			
	Beta coefficients	Lower bound	Upper bound	*t*-value	*p*-value
(Constant)	15.989	8.813	23.165	4.394	<.001
Age (years)	.053	−.140	.246	.542	.589
Sex = Male	−1.715	−3.188	−.242	−2.297	.023
Year of graduation ≥2011	3.482	1.388	5.575	3.280	.001
Specialty	−.199	−.561	.163	−1.082	.281
Working place/sector	−.427	−1.014	.159	−1.437	.152
Prefers Hybrid (Online + Onsite (face-to-face)) learning	3.189	.932	5.446	2.786	.006
Prefers Onsite (face-to-face) learning	3.965	1.994	5.937	3.967	<.001

DV = Mean perceived usefulness of optics courses. Model *R* = 45.1%, adjusted *R*-squared = 17.5%.

## Discussion

Optics-based courses are traditionally believed to form the backbone of optometry curriculum [17]. However, it has recently been argued that biomedical courses should take precedence over optics ones. This study used a cross-sectional survey to obtain feedback from optometrists regarding the usefulness of optics courses in their professional endeavours. Our analysis revealed that the highest perceived ranking was assigned to the statement ***"I believe that optics courses should be taught in the optometry curriculum," with a*** substantially high relative importance index = 75.1%. Furthermore, the aspect ***"The practitioner licence exam included questions from many areas of these optics courses,"*** occupied the second usefulness rank among optics courses, with a substantial relative importance weight of 70.8%. As optometrists must pass several scheduled examinations to obtain a licence to practice the profession legally, these findings are highly relevant and reflect the high utility of the optometry curriculum. Notably, the third rank on the perceived usefulness of optics courses was ascribed to ***"The educational material in these courses I received was relevant to my career,"*** with a significant relative importance weight of 70.4%, again reflecting the high relevance and utility of the optometry curriculum.

The lowest agreement scores were assigned to the lecturer's ability (61.4%) and the enjoyment of learning optics courses (67%). It is known that students face problems in learning science courses [32], and students' low achievement in science courses is based on the abstract scientific facts that these courses contain and how they are delivered and explained to students [33–35]. One factor that could help in this regard is the inclusion of practical examples in textbooks to help students understand the optical concepts relevant to eye clinics. Furthermore, lab activities related to optics courses should be included to help students understand the conceptual components of optics. Well-designed optical laboratories enhance and help students understand the abstract principles and concepts of the materials covered in these courses. Unfortunately, the optics laboratory component of the theoretical lectures was not one of the questions asked by respondents in this research. Our results also suggest that the perceived usefulness of optics courses was higher among post-2011 graduates than among those who graduated before. This could be explained by the former's better experience of being taught optics courses and the fact that optics laboratories have become well equipped with optical equipment since 2011, which significantly supports the comprehension of theoretical optics courses.

This study has some limitations related to the online nature of the study. The study used an online survey, which may have resulted in self-report bias, as respondents are often biased when reporting their personal experiences. Recall bias is also possible, considering the time-lapse. Furthermore, there is no record of the academic achievement of the respondents in these optics courses, which might have helped to examine the association between the perception of the respondents in these optics courses and their academic achievement. Furthermore, we did not examine whether the curriculum was altered after its introduction in any of the years. Finally, while we discovered that some characteristics, such as age, sex, and mode of education, have an impact on the outcome, we currently lack data to explain the underlying determinants. In the future, multicenter investigations should be conducted to address these limitations and better elucidate the findings.

The questions used in this study cover Kirkpatrick levels 1–3, reflecting the high functional utility of optics in the curriculum. Question 1 of the questionnaire used in the current work belongs to Kirkpatrick level 3, as this question addresses whether the knowledge gained from the optics course is applied to practice. Questions 2 and 7 belong to Kirkpatrick Level 2, as they indicate that the respondents have gained knowledge from the optics course, which they have utilised to obtain practitioner licences and other career objectives. Questions 3, 4, and 5 fit Kirkpatrick levels 1 and 2, as they capture the respondents' reactions to the need and suitability of the optics course. Future studies are needed to examine the impact of optics in the optometry curriculum on improving patient care (Kirkpatrick Level 4) [28]. Other aspects of teaching optics courses should also be studied in future work, especially in laboratories associated with these optics courses, including their readiness and enhancement to support theoretical optics courses.

## Conclusions

This study assessed the perceptions of the usefulness of optics courses and their relevance to optometry professionals from the perspective of optometrists in Jordan. The perception of importance level ranged from the highest rank in terms of the usefulness of these courses to be included in the curriculum and their value to the practice of optometrists, regardless of their specialties and workplace. The least important factor was the interest of optometrists in these courses throughout their studies. Optometrists within various specialties and work sectors may not necessarily differ significantly in their perception of the usefulness of optics courses, suggesting that they may have needed optics courses almost constantly, regardless of their specialty.

## Data Availability

The datasets generated and/or analysed during the current study are available from the corresponding author on request.
